# Titanium dioxide in face powders and eyeshadows: Developing an analytical methodology for accessing customer safety

**DOI:** 10.1111/jocd.16422

**Published:** 2024-06-19

**Authors:** Diana Amorello, Marzia Ferrara, Santino Orecchio

**Affiliations:** ^1^ Department of Science and Technology Biological, Chemical and Pharmaceutical University of Palermo Palermo Italy

**Keywords:** analytical method, cosmetics, eyeshadows, powders, titanium oxide

## Abstract

**Background:**

Titanium dioxide (TiO_2_), a white powder, represents the opacifier used in many products, including drugs, foods, cosmetics, paints, and dyes.

**Method:**

The Uv‐Vis spectrophotometry was a particularly suitable technique to quantify TiO_2_ in the solutions obtained from cosmetics. In this work, we determined the TiO_2_ content in a total of 88 samples of eye shadows and face powders of different brands and costs. Before to analyse the samples, we developed the mineralization and analysis method, in fact, fusion with potassium bisulphate would be very laborious because it must be carried out on one sample at a time and requires very long times, instead, the mineralization with the acid mixture and the aid of microwaves allowed us to solubilize six samples at the same time within 45 min.

**Results:**

From the results obtained, we can state that the highest concentrations of TiO_2_ are found in the eyeshadows with a maximum value of 36% in a blue eyeshadow.

## 1 INTRODUCTION

The term cosmetic defines any substance or mixtures intended to be applied to the external surfaces of the human body (epidermis, hair, nails, lips, etc.), for the purpose of cleaning, perfuming, changing appearance, protecting, keeping in good condition, or correcting odors bodily. The following products are considered cosmetics: creams, lotions, gels and oils for the skin, beauty masks, foundations and in general all make‐up products, perfumes, preparations for baths and showers, for depilation, deodorants and antiperspirants, dyes and other products for hair, products for shaving and for the care of teeth, mouth, nails and sunscreen or self‐tanning products.^1^

The cosmetics history begins with the history of man; in fact, the art of cosmetics developed among the first civilizations of the Middle Eastern area of the Mediterranean. Historical proof are the Sumerian statuettes showing how eye makeup was in use. In Egypt, for this purpose Kohl or Kajal was used, a powder composed mainly of galena, malachite, antimony, and animal fat, obtained by mixing the ash of almond shells with lead and copper salts, used for eye make‐up, to darken the eyelids and outline the eye contour using a special felt stick. Cosmetics have always been widely consumed products as, generally, their use is aimed at improving the appearance and to protect the external surfaces of the human body, therefore, consumers do not consider them possible causes of health risk. Cosmetics, defined by Regulation (EC) 1223/2009 of the European Parliament and by the Council of 30 November 2009 on cosmetic products,^1^ consist of multiple natural or synthetic chemical substances which in some cases can cause the onset of various events negatives such as, for example, those allergic. The potential risks associated with the use of cosmetics may depend on various factors, for example, the presence of allergenic components, contaminants, or degradation substances due to incorrect storage. Some heavy metals, in the past, have been used as components of cosmetic products, such as lead acetate, used as a hair dye and red cinnabar (mercury sulfide) already used in the times of the Roman Empire. In the 1930s, the thallium contained in depilatories caused cases of serious and sometimes lethal intoxications. In the 1950s and 1960s, zirconium‐containing deodorants caused cases of inflammatory allergic skin reactions in consumers in Europe and the United States.^2^, ^3^, ^4^

Every day, people are exposed to several hazardous substances (metals, PAHs, phthalates, pesticides, etc.) through a wide range of routes.^5^, ^6^, ^7^, ^8^, ^9^ Although atmospheric emissions tend to be the greatest cause for concern in terms of human exposure and health;^7^ however, other less obvious sources of exposure^9^, ^10^ must be considered, including the use of cosmetic, pharmaceutical and tattoo products.^10^

Cosmetics can be an important way of exposure to dangerous chemicals since their components come into direct contact with the epidermis and mucous membranes. The greater presence of women in some professional activities, related to esthetics, leads to their greater exposure to the components of these products. The Cosmetica Italia Association reported in 2018 that daily women use meanly fourteen cosmetics against seven of men.^11^

In women, there is a greater risk that use and exposure to cosmetics lead to adverse effects, some of which potentially dangerous, especially in the case of the use of adulterated, falsified or, in any case, products that do not comply with the directives. In fact, even if cosmetics are strictly regulated by European legislation, various substances such as compounds of lead, mercury, phthalates and parabens, present frequently in counterfeit products or sold in street stalls and markets, cause concern for their possible effects on health, particularly for women of reproductive age.^10^ Irritating, allergic and photo dermatitis are among the most common negative effects associated to the cosmetic contact; all manifestations with a strong component linked to gender, for example, contact dermatitis caused by nickel compounds, have a female/male occurrence ratio of 3:1, due both to the more frequent use of such products and to the greater susceptibility to the disease, with typical localizations on the eyelids and armpits, due to the allergens contained in make‐up and deodorant.^10^

Metals and their compounds, which can be classified as cosmetic contaminants or pigments/opacifiers themselves, have been associated with cutaneous pathologies.^12^, ^13^, ^14^, ^15^, ^16^ In the past, red tattoo inks, containing mercury and those yellow, containing cadmium were considered to be the most hazardous^1^ In Magna Graecia, it was customary to use a white pigment, white lead, which gave to the skin an accentuated white color to the women body.

Titanium dioxide (TiO_2_), calcium and magnesium carbonate, calcium sulfate, aluminum silicates, aluminum oxide and hydroxide are the most commonly opacifiers used in cosmetic products. TiO_2_, a white powder, known as E171 in the European coding of food additives, represents the opacifier used in many products, including drugs, foods, cosmetics, paints, dyes, etc.^17^ Although, for many time, the use of TiO_2_ was considered safe, based on new in vitro and in vivo researches, many doubts have been recently raised.^17^

Titanium dioxide, upon the exposure to UV light, even in aqueous matrices, was shown to catalyze the production of reactive oxygen species, such as superoxide anion, hydroxyl radical and singlet oxygen.^18^ Additional in vitro experiments demonstrated that, even in the absence of photo activation, nanoscale particles (<200 nm) would cause oxidative DNA damage, lipid peroxidation, micronucleus formation, production of H_2_O_2_, and nitric oxide.^19^

The control concerning the TiO_2_ in cosmetics is very difficult because most of the products are purchased online, often from small companies located in areas where the legislation is more permissive than the European one. To the best of our knowledge, there are few data on the concentration of TiO_2_ in in face powders and eyeshadows,^19^, ^20^ in particular for those sold online or by makeshift hawkers. Considering what has been said previously, during this research has been optimized a simple, fast, and inexpensive method to quantify titanium in complex matrices such as those of the different types of cosmetics which can has quality parameters (linearity, reproducibility, accuracy, limit of detection, and limit of quantification) suitable for the purpose. In particular, samples of eyeshadows and powders were taken into consideration since they are cosmetic products that have very different compositional characteristics. For the titanium quantification, we have used a UV–Vis spectrophotometric method.

## 2 RESULTS AND DISCUSSION

Similarly, to the tests carried out as in previous researches,^21^, ^22^, ^23^ the LOD and LOQ values for TiO_2_ are 0.04% and 0.13%, respectively. To evaluate the accuracy, we carried out the analysis three times, using the developed method, of four solutions prepared starting from known quantities of metallic titanium having concentrations of 2.65, 5.30, 6.45, and 12.9 ppm, obtaining on average concentrations that differed by more or less than 4% from the theoretical value. To evaluate the precision of the whole method, calculated as the relative standard deviation (RSD%), we carried out the analysis, as previously described, of five solutions prepared starting from known quantities of two different samples, obtaining RSD between 3% and 9%. To verify precision, we repeated the complete analysis of six of the randomly chosen samples five times. On average, the precision was ±6%, a value which for the purposes of the method is quite satisfactory. Considering all the samples taken into consideration, the determination coefficient (r^2^) of calibration curves were between 0.9939 and 1 for face powders, while for eyeshadows it was between 0.9893 and 1, indicating good linearity between absorbance and concentration. For the element investigated these values were considered satisfactory.

In the eyeshadow samples the concentrations, expressed as TiO_2_, are between 0.04% and 36%. From the results obtained (Table 1 and Figure 1) we can state that the highest concentrations of TiO_2_ are found in the eyeshadows with a maximum value of 36% in the sample No. 5 (blue ES). Taking into account that the most important make‐up manufacturers establish that the optimal percentages, in relation to the good yield of the products, are between 1% and 5%, we can state that in the majority of the samples taken into consideration this range is not respected.^24^ As regards powders, the highest percentage (20%) was found in sample No. 30 (Pa2) (Table 2, Figure 2). Considering that TiO_2_ is used as a white pigment, we expected that the highest concentrations would be mainly contained in the samples of light powders and eyeshadows; this assumption of ours was disproved by the experimental results. For the most part, it is the samples with the darkest shades that contain the most titanium. This experimental evidence can be associated with the fact that darker shades must have greater coverage/compactness and greater durability, therefore requiring a greater quantity of TiO_2_ from the manufacturing companies. According to the recent decision of the SCCS and with the entry into force of the new regulation on TiO_2_,^24^, ^25^, ^26^ almost all the samples analyzed by us would be considered unmarketable, since they exceed the concentration of 1%.

**TABLE 1:** Titanium oxide concentration (%) in the eyeshadows samples.

No.	Sample	Color	TiO_2_%	No.	Sample	Color	TiO_2_%
1	T	Black	1.7	23	bD	Heavenly	6.6
2	DD	Black	4.7	24	cD	Heavenly	4.9
3	ES	Heavenly	16	25	D	White	2.5
4	ES	Orange	1.8	26	D	Gold	5
5	ES	Blue	36	27	aP	Violet	6.9
6	Baby P	Violet	1.9	28	R	White	2.6
7	Baby P	Pink	2.3	29	D	White	2.5
8	Baby P	Gray	4.9	30	BN	Black	4.2
9	baby P	Yellow	9.3	31	aLA	Pink	11
10	Baby V	Orange	1.3	32	LA	Fuchsia	20
11	AK	Gray	0.04	33	bD	Brown	7.8
12	P	Ivory	11	34	cD	Brown	4.4
13	P	Violet	5.1	35	P	Black	5.2
14	P	Pink	8.9	36	P	White	3.5
15	P	Heavenly	8.1	37	Kc	Green	11
16	P1	Gray	4.5	38	Ka	Green	8.9
17	Baby V	Yellow	1.3	39	Kc2	Green	13
18	Baby V	Violet	3.3	40	P	Indigo	6.3
19	Baby V	Gray	1.3	41	K	Black	2.5
20	D	Black	4.3	42	cK	Brown	11
21	aD	Green	6.6	43	LA	Indigo	5.1
22	aD	Heavenly	4.8	44	DI	Gold	17

**FIGURE 1:**
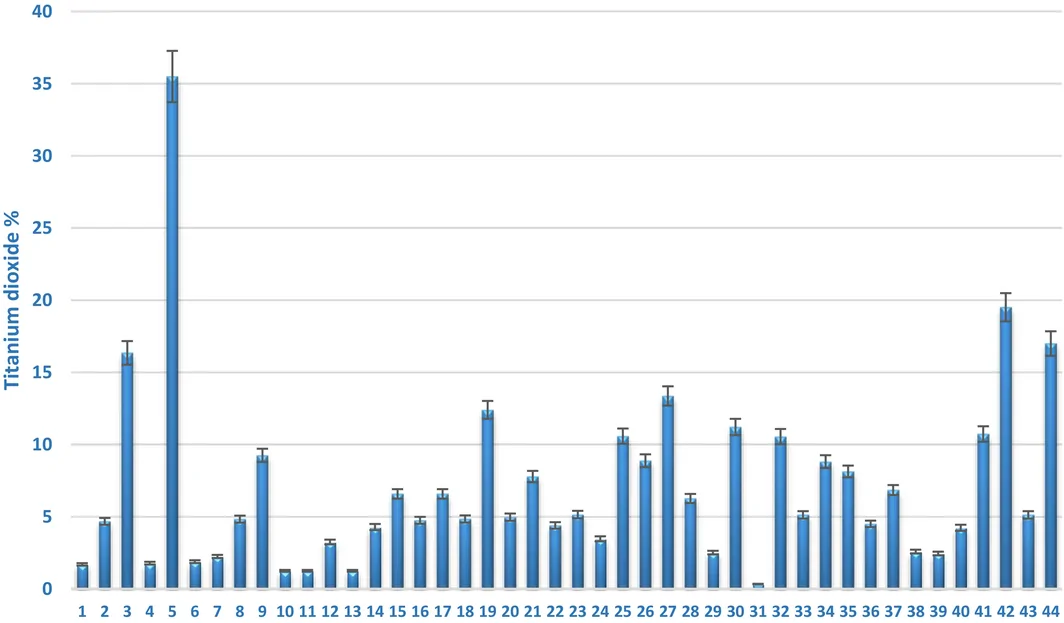
Titanium oxide concentrations in eye shadows.

**TABLE 2:** Titanium oxide concentrations in powder samples.

No.	Sample	TiO_2_%	No.	Sample	TiO_2_%
1	Sua	5.4	23	Da1	2.8
2	SUb	8.9	24	Dc	6.7
3	SUc	2.3	25	Da2	3.7
4	Ara	2.9	26	Dc4	5.6
5	Arb	4.5	27	Da2	3.7
6	ISa	0.4	28	Db	7.7
7	Baby P	4.7	29	Pb	3.3
8	Baby Va	4.5	30	Pa	20
9	Baby Vb	1.8	31	Pa2	<0.04
10	Ka	<0.04	32	Pb2	0.9
11	Kb	<0.04	33	Pc2	1.5
12	Kc2	6.6	34	Aa2	5.3
13	INCa	4.7	35	Ab2	8.0
14	Aa	2.6	36	Ac2	5.1
15	Ab	<0.04	37	Wa	1.1
16	Ac	2.2	38	Ka3	12
17	BO	<0.04	39	Kb3	7.9
18	Da3	3.4	40	DGb	2.9
19	Db3	4.6	41	Dga	2.9
20	Dc3	3	42	BNb	1.5
21	Vya	8.7	43	UNIFa	0.5
22	DIb	8.3	44	CHa	<0.04

**FIGURE 2:**
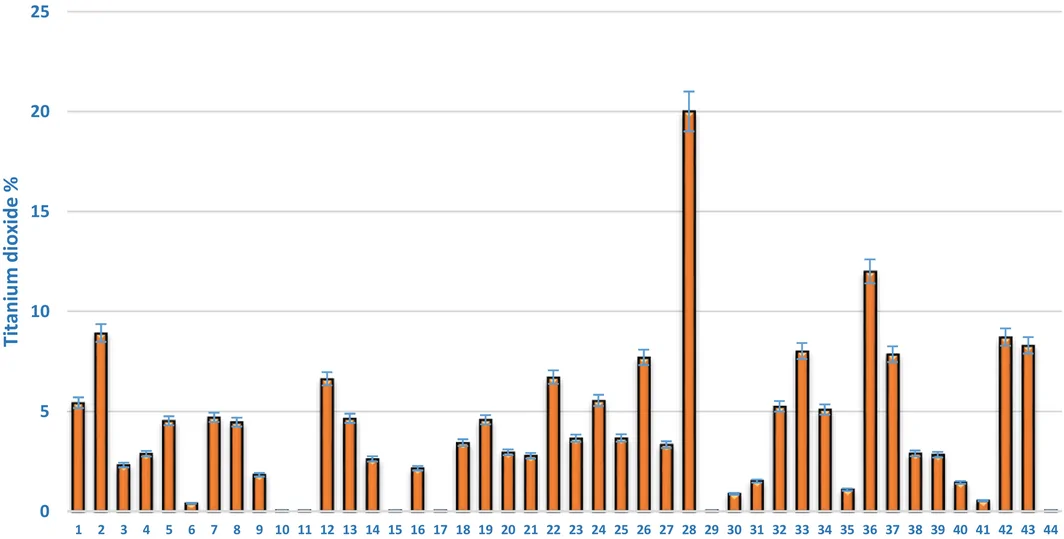
Titanium oxide concentrations in powder samples.

Furthermore, manufacturing companies should label cosmetics containing TiO_2_, not only as already done with the CI 77891 identification code, but also reporting the specific attention code: EUH212: (Warning! Dangerous respirable dust may form if used). Do not breathe dust.^27^

Furthermore, being now classified as a class 2 carcinogen by inhalation, TiO_2_ can no longer be used in the form of a nano material, while if the aforementioned pigment has a particle diameter greater than 10 μm it can be used in the 1%–5% range.

## 3 CONCLUSION

The Uv–Vis spectrophotometry was a particularly suitable technique for our purposes because it is above all useful in the analysis of the solutions obtained from the cosmetic products because from color to color and from brand to brand, the samples are very heterogeneous in composition. Considering the standard deviation values on found concentrations we can affirm that the method provides accurate results and therefore suitable for the determination of the investigated metal in cosmetic samples.

In this work, we determined the TiO_2_ content in a total of 88 samples of eye shadows and powders of very different brands and costs. Before analyzing the samples, as already mentioned, we developed the mineralization and analysis method; in fact, fusion with potassium bisulfate would be very laborious because it must be carried out on one sample at a time, and requires very long times; instead, the mineralization with the acid mixture and the aid of microwaves allowed us to bring six samples into solution at the same time within 45 min. Furthermore, as described in the experimental part, we evaluated the quality parameters that are quite satisfactory for the purpose of the study. The spectrophotometric technique was simple, fast and free from interference, unlike other much more complex and expensive instrumental techniques.

Regarding the presence of TiO_2_ in the samples, we can state that from the data obtained, the highest concentrations (on average 7%) of TiO_2_ were found in the most expensive face powders, while, with some exceptions, in the less expensive products, the quantities of TiO_2_ are lower, around 4%. This could be associated with the fact that using TiO_2_ has a significant cost, so for a small company, using less TiO_2_ can lead to economic savings. As regards eyeshadows, the situation appears similar to what was observed for face powders; in fact, apart from some exceptions, in the cheaper products the average percentage of TiO_2_ is around 12%, while in the more expensive ones the average concentration is 35%. As regards the samples in the medium price range, the ones we mainly took into consideration, the situation appears similar for both face powders and eye shadows, with a concentration of around 9% of TiO_2_. It is worth noting that, in most of the cosmetic products analyzed, the concentration of TiO_2_, in light of recent legislation, is higher than the permitted limits. From the results obtained, the danger of many cosmetic products, even those of high cost, is highlighted. We can therefore hypothesize that the circulation of illegal products on the market, in this case, is due to the fact that the legislation is recent and unclear and the checks are generally carried out on the products available in perfumeries and shopping centers but, certainly, not on those sold online or in neighborhood markets.

## 4 EXPERIMENTAL SECTION

### 4.1 Quality control and quality assurance

The choose of procedural blank in trace analytical determination is basic to assess contamination and matrix interferences. The detection (LOD) and quantification (LOQ) limits of the method, as in other researches^21^, ^22^, ^23^ was established by measure of 10 procedural blanks. To obtain LOD and LOQ, calibration blank or ultrapure water were not used because they could not estimate matrix interferences. Otherwise, to the best of our knowledge, a cosmetic product without analita was not available at present. For these reasons, the procedural blank was obtained by subjecting ten different aliquots of procedural blank (2 mL of HNO_3_, 2 mL of H_2_O_2_), to the entire mineralization procedure.

### 4.2 Laboratory equipment

All glassware and sample containers were thoroughly washed with hot HNO_3_ 3% solution followed by rinsing with double‐distilled purified water and acetone (analytical grade), respectively. These were finally kept in the oven at 110°C overnight. To avoid contaminations of samples, different glassware and pipettes were used for standards and for solutions obtained from cosmetic samples.

### 4.3 Cosmetic samples

Some of the samples used in this study were provided by colleagues and friends, many were purchased in shops not suitable for such sale or at stalls in neighborhood markets, while the most expensive products were purchased in pharmacies. In total, the samples taken into consideration are: 44 face powders and 44 eyeshadows. Table 3 shows, in ascending order of price, the acronyms attributed to the eyeshadow samples (to avoid reporting the brand) and their color:

**TABLE 3:** Acronyms attributed to the eyeshadow samples.

No.	Sample	Color	No.	Sample	Color	No.	Sample	Color
1	T	Black	17	BabyV	Giallo	33	bD	Brown
2	DD	Black	18	BabyV	Violet	34	cD	Brown
3	ES	Heavenly	19	Baby V	Grigio	35	P	Black
4	ES	Orange	20	D	Black	36	P	White
5	ES	Blue	21	aD	Green	37	Kc	Green
6	Baby P	Violet	22	aD	Heavenly	38	Ka	Green
7	Baby P	Pink	23	bD	Heavenly	39	Kc2	Green
8	Baby P	Gray	24	cD	Heavenly	40	P	Indigo
9	baby P	Yellow	25	D	White	41	K	Black
10	babyV	Orange	26	D	Gold	42	cK	Brown
11	aK	Grigio	27	aP	Violet	43	LA	Indaco
12	P	Panna	28	R	White	44	DI	Gold
13	P	Violet	29	D	White			
14	P	Pink	30	BN	Black			
15	P	Heavenly	31	aLA	Pink			
16	P1	Gray	32	LA	Fuchsia			

Powders

- a, b c: indicate the shades from the lightest (a = light pink) to the darkest (c = brown);
- 1, 2, 3: same brand, different palette.

### 4.4 Preparation of the titanium standard solution

189.9 mg of 99% metallic Ti, weighed on the analytical balance (±0.1 mg), were solubilized in 98% H_2_SO_4_. The solution obtained was brought to 100 mL with double‐distilled water. The high concentration (1899 ppm) of this standard solution will allow very small volumes to be added to the solutions obtained from the mineralization of cosmetic samples to use the standard addition method in spectrophotometric analyses.

### 4.5 Sample mineralization

Considering the few bibliographic data and some preliminary studies, we decided to use cosmetic approximately 100 mg of samples. The classic method for solubilizing samples containing TiO_2_ is that of alkaline melting. In practice, the finely powdered sample mixed in a 1:1 ratio with potassium bisulfate is heated in a platinum crucible until it completely melts. The cooled mixture must be solubilized in HCl.^28^ Considering the very refractory nature of TiO_2_ and the complexity of the above method, for the mineralization of the samples, we use 2 mL of 98% H_2_SO_4_, 2 mL of 85% H_3_PO_4_, 1 mL of 49% HF, and 0.5 mL of 70% HNO_3_ in a microwave mineralizer (Milestone model MLS‐1200 Mega; Milestone Laboratory Systems, Italy). The instrumental conditions used for the microwave digestion were: 1 min at 250 W, 1 min at 0 W, 5 min at 250 W, 5 min at 450 W, 4 min at 600 W, and 5 min at 250 W. After mineralization, the solutions were brought almost dry using a heating pad and Teflon containers to eliminate HF and HNO_3_, which interfere with the analysis method.^29^ After this procedure, the solution was brought to volume (generally to 50 mL) with water and sulfuric acid, making sure that the concentration of the latter was between 1.5 N and 3.5 N, in order to maintain the solution all possible oxides and hydroxides.

### 4.6 Spectrophotometric analysis

The presence of titanium in the solutions obtained as previously described is indicated by yellow color depending on the concentration due to the (Ti [H_2_O_2_])^+4^ complex.^28^ To perform the analysis, the spectrum of the sample solution obtained from the previous treatment was recorded from 300 to 600 nm (Jasco UVIDEC—610 spectrophotometer) to which 0.1 mL of 30% H_2_O_2_ was added. The absorbance was measured against a blank consisting of the same solution acidified with H_2_SO_4_ but without hydrogen peroxide. For quantification, as already mentioned, the standard addition method was used, we added 50 μL aliquots of the standard solution (1899 ppm) from time to time to 6 mL of sample solution. The absorbance value was determined at 400 nm. The values obtained were plotted as a function of concentration. By extrapolating the straight line thus obtained on the negative axis of concentrations, it was possible to calculate the concentration of titanium in the solution obtained from mineralization after any dilution.^28^ Figure 3, as an example, shows the spectra of two samples (powder and eyeshadow) obtained with the standard addition method.

**FIGURE 3:**
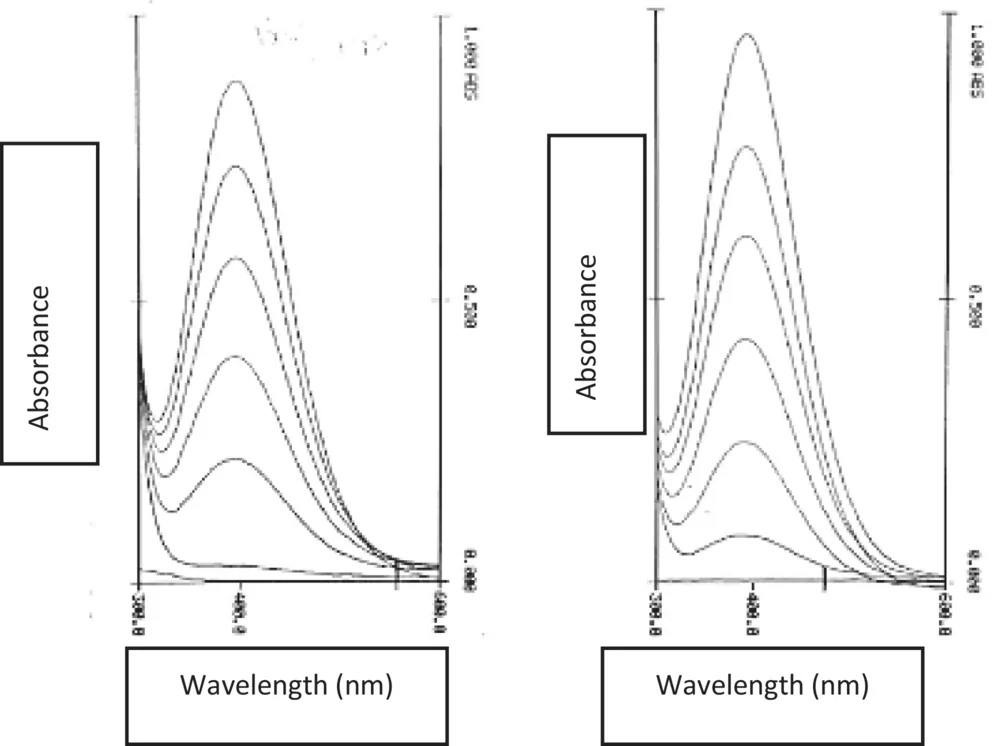
UV–Vis spectra of a powder and eyeshadow samples obtained with the standard addition method.

## ETHICS STATEMENT

This study does not involve any human participants and was conducted entirely on samples. Therefore, no ethics approval or informed consent was required.

## Data Availability

Research data are not shared.

## References

[1] Gazzetta ufficiale dell'Unione Europea (22.12.2009) L 342/59 REGOLAMENTO (CE) n. 1223/2009 DEL PARLAMENTO EUROPEO E DEL CONSIGLIO del 30 novembre 2009 sui prodotti cosmetici.

[2] Barbaud A , Lafforgue C . Risks associated with cosmetic ingredients. Ann Dermatol Venereol. 2021;148:77‐93. 10.1016/j.annder.2020.04.027 33642039

[3] Rapporti ISTISAN 19/24; Istituto Superiore Sanità. 2019.

[4] Alimonti A , Bocca B , Buratti FM , Fabbri E , Testai E . Metalli in prodotti cosmetici: procedure raccomandate per la determinazione e valutazione dei rischi per la salute. Istituto Superiore di Sanità; 2009.

[5] Orecchio S , Amorello D , Barreca S . Wood pellets for home heating can be considered environmentally friendly fuels? Heavy metals determination by inductively coupled plasma‐optical emission spectrometry (ICP‐OES) in their ashes and the health risk assessment for the operators. Microchem J. 2016;127:178‐184.

[6] Orecchio S , Barreca S , Indelicato R . Determination of selected phthalates by gas chromatography–mass spectrometry in personal perfumes. J Toxicol Environ Health A. 2015;78:1008‐1018. 10.1080/15287394.2015.1021433 26262443

[7] Amorello D , Barreca S , Gulli E , Orecchio S . Platinum and rhodium in wine samples by using voltammetric techniques. Microchem J. 2017;140:229‐245.

[8] Orecchio S , Fiore M , Barreca S , Vara G . Volatile profiles of emissions from different activities analyzed using canister samplers and gas chromatography‐mass spectrometry (GC/MS) analysis: a case study. Int J Environ Res Public Health. 2017;14:195. doi:10.4490/ijerph14020195 PMC5334749 28212294

[9] Orecchio S , Indelicato R , Barreca S . Determination of selected phthalates by gas chromatography–mass spectrometry in mural paintings from Palermo (Italy). Microchem J. 2014;114:187‐191.

[10] Di Gaudio F , Amorello D , Ferrara M , Orecchio S , Orecchio S . Heavy metals in tattoo inks: developing an analytical methodology for accessing customer safety. ChemistrySelect. 2023;8:202300986.

[11] https://www.cosmeticaitalia.it/centro‐studi/Beauty‐Report‐2019/

[12] Arshad H , Mehmood MZ , Shah MH , Abbasi AM . Evaluation of heavy metals in cosmetic products and their health risk assessment. Saudi Pharm J. 2020;28:779‐790. 10.1016/j.jsps.2020.05.006 PMC7335825 32647479

[13] Ababneh FA , Al‐Momani IF . Assessments of toxic heavy metals contamination in cosmetic products. Environ Forensic. 2018;19(2):134‐142.

[14] Al‐Saleh I , Al‐Enazi S . Trace metals in lipsticks. Toxicol Environ Chem. 2011;93(6):1149‐1165.

[15] Bocca B , Pino A , Alimonti A , Forte G . Toxic metals contained in cosmetics: a status report. Regul Toxicol Pharmacol. 2014;68(3):447‐467. 10.1016/j.yrtph.2014.02.003 24530804

[16] Łodyga‐Chruścińska E , Sykuła A , Więdłocha M . Hidden metals in several brands of lipstick and face powder present on polish market. Cosmetics. 2018;5:57. doi:10.3390/cosmetics5040057

[17] Winkler HC , Notter T , Meyer U , Naegeli H . Critical review of the safety assessment of titanium dioxide additives in food. J Nanobiotechnology. 2018;16:51. 10.1186/s12951-018-0376-8 PMC5984422 29859103

[18] Konaka R , Kasahara E , Dunlap WC , Yamamoto Y , Chien KC , Inoue M . Ultraviolet irradiation of titanium dioxide in aqueous dispersion generates singlet oxygen. Redox Rep. 2001;6(5):319‐325. 10.1179/135100001101536463 11778850

[19] Gurr J‐R , Wang ASS , Chen CH , Jan KY . Ultrafine titanium dioxide particles in the absence of photoactivation can induce oxidative damage to human bronchial epithelial cells. Toxicology. 2005;213(1–2):66‐73. 10.1016/j.tox.2005.05.007 15970370

[20] Palugan L , Spoldi M , Rizzuto F , et al. What's next in the use of opacifiers for cosmetic coatings of solid dosage forms? Insights on current titanium dioxide alternatives. Int J Pharm. 2022;616:121550. 10.1016/j.ijpharm.2022.121550 35131353

[21] Barreca S , Orecchio S , Orecchio S , Abbate I , Pellerito C . Macro and micro elements in traditional meals of Mediterranean diet: estimated intake by the population. J Food Compos Anal. 2023;123:105541.

[22] Amorello D , Orecchio S , Barreca S , Orecchio S . Voltammetry for monitoring platinum, palladium and rhodium in environmental and food matrices. ChemistrySelect. 2023;8:e202300200.

[23] Barreca S , Orecchio S , Orecchio S , Abbate I , Pellerito C . Macro and micro elements in traditional meals of Mediterranean diet: estimated intake by the population. J Food Compos Anal. 2023;123:105541.

[24] Selleri R , Botrè C , Orzalesi G . Chimica e tecnologia dei prodotti cosmetici. 1977 37. Ragno.

[25] Titanium dioxide. Website ECHA, echa.europa.eu/it/registry‐of‐clh‐intentions‐until‐outcome/−/dislist/details/0b0236e18075da 38

[26] Scientific Committee On Consumer Safety (SCCS) . Request for a scientific opinion on Titanium dioxide (TiO2) (CAS/EC numbers 13463–67‐7/236–675‐5, 1317‐70‐0/215–280‐1, 1317‐80‐2/215–282‐2) used in cosmetic products.

[27] Regolamento (CE) n.1223/2009 del Parlamento europeo e del Consiglio del 30 novembre 2009 sui prodotti cosmetici.

[28] Mendham J , Denney RC , Barnes D , Thomas MJK , eds. Vogel's Textbook of Quantitative chemical analysis. 1995.

[29] Holler FJ , Crouch SR . Fondamenti di Chimica Analitica di Skoog e West. 2015.

